# Do Self-Expanding Metal Stents as a Bridge to Surgery Benefit All Patients with Obstructive Left-Side Colorectal Cancers?

**DOI:** 10.1155/2019/7418348

**Published:** 2019-02-03

**Authors:** Jun-rong Zhang, Ping Hou, Tian-ran Liao, Yong Wei, Xian-qiang Chen, Bing-qiang Lin

**Affiliations:** ^1^Department of General Surgery (Emergency Surgery), Fujian Medical University Union Hospital, No. 29 Xinquan Road, Fuzhou, 350001 Fujian, China; ^2^Immunotherapy Institute, Fujian Medical University, No. 1 Xuefu Bei Road, Fuzhou, 350122 Fujian, China

## Abstract

**Background:**

Self-expanding metal stents (SEMS) have been increasingly used in patients with obstructive left-sided colorectal cancer (OLCC); however, stent-specific complications (e.g., perforations) might worsen the long-term survival outcome. Strict indication needed to be identified to confirm the benefit subgroups. This study was designed to explore the indication for emergency surgery (ES) and SEMS in patients with OLCC and to suggest optimal strategies for individuals.

**Methods:**

After propensity score matching, 36 pairs were included. Perioperative and long-term survival outcomes (3-year overall survival (OS) and 3-year disease-free survival (DFS)) were compared between the ES and SEMS groups. Independent risk factors were evaluated among subgroups. Stratification survival analysis was performed to identify subgroups that would benefit from SEMS placement or ES.

**Results:**

The perioperative outcomes were similar between the SEMS and ES groups. The 3-year OS was comparable between the SEMS (73.5%) and ES (60.0%) groups, and the 3-year DFS in the SEMS group (69.7%) was similar to that in the ES group (57.1%). The pT stage was an independent risk factor for 3-year DFS (*p* = 0.014) and 3-year OS (*p* = 0.010) in the SEMS group. The comorbidity status (*p* = 0.049) independently affected 3-year DFS in the ES group. The 3-year OS rate was influenced by the cM stage (*p* = 0.003). Patients with non-pT4 stages in the SEMS group showed obviously better 3-year OS (95.0%) than the other subgroups. The 3-year OS rate was 36.4% in the ES group when patients had a worse comorbidity status than their counterparts.

**Conclusion:**

SEMS might be preferred for patients of obstructive left-sided colorectal cancer in the “high-operative risk group” with existing comorbidities or those without locally advanced invasion, such as the non-pT4-stage status.

## 1. Introduction

Obstructive left-sided colon cancer (OLCC) is the primary cause of acute malignant colonic obstruction, with an incidence rate ranging from 58% to 80% [1–3]. Although several strategies, including emergency Hartmann's procedures, emergency surgery (ES) with stoma construction followed by a staged procedure, ES with intraoperative irrigation or manual decompression, and self-expandable metal stents (SEMS) as a bridge to surgery (BTS), have been introduced in the treatment of OLCC, the best option remains elusive [4–9]. Since SEMS placement with elective surgery could improve nutrition and physical status and alleviate colonic distention, thereby increasing the likelihood of primary anastomosis and reducing the risk of colostomy, it has been widely advocated as the first choice for OLCC treatment [6, 10–12]. However, several randomized controlled trials and multicenter prospective studies recently ended prematurely due to the extremely high rates of stent-related perforation, which worsened the long-term survival results and increased the risk of recurrence [13–16]. These adverse effects hinder the widespread use of SEMS in the treatment of OLCC. The indication of SEMS or ES for the management of OLCC remains undetermined. Due to similar survival outcomes to ES, SEMS placement was recommended for OLCC treatment, without elaborate indication, in the latest NCCN guideline [17]. The European Society of Gastrointestinal Endoscopy (ESGE) identified SEMS placement as the superior choice for patients in the “high-risk” group, which is defined as older patients and/or those with the American Society of Anesthesiologists (ASA) scores greater than 3. Conversely, ES was deemed suitable for patients in the “low-risk” group [18, 19].

This study was designed to identify the risk factors for 3-year overall survival (OS) and 3-year disease-free survival (DFS) for ES and SEMS placement. We primarily explored the indications for the use of alternative methods in OLCC patients based on the stratification analysis of long-term survival.

## 2. Materials and Methods

### 2.1. Patient Population

All patients (*n* = 162) who underwent surgery for obstructive colorectal cancer (OCC) at the Department of Emergency Surgery at Fujian Medical University Union Hospital from January 2008 to October 2014, with all operations performed by a single surgical group, were retrospectively evaluated. The study protocol was approved by the Institutional Review Board of our hospital, and all patients provided written informed consent for surgery.

### 2.2. Classification Criteria

**Figure 1:**
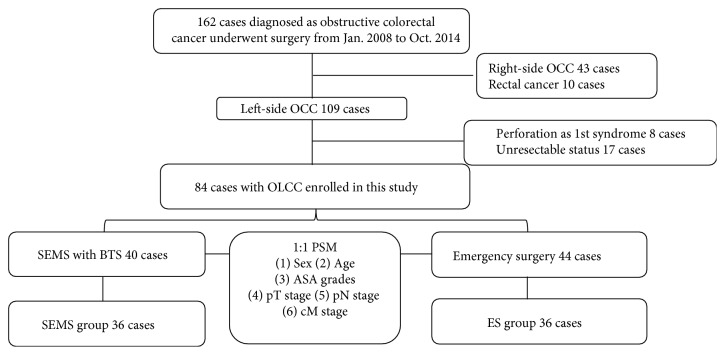
Patient inclusion flowchart. OCC: obstructive colorectal cancer; SEMS: self-expandable metal stent; BTS: bridge to surgery; ES: emergency surgery; PSM: propensity score matching.

Patients who primarily manifested complete or incomplete bowel obstructive syndrome were enrolled in this study. All diagnoses of left-sided colorectal obstruction were confirmed by emergency abdominal computed tomography (CT), and diagnoses of malignancy were confirmed by pathological examination; all stage IV patients underwent radical resection for distant metastasis lesions. Seventy-eight patients were excluded from this study, including 43 with right-side OCC, 10 with rectal cancer, 8 with acute peritonitis with perforation, and 17 who underwent surgery without radical dissection intent. Detailed information on our classification criteria is shown in Figure 1.

### 2.3. Surgical Procedures

Isoperistaltic lavage or manual decompression for intraoperative clearance was performed as described previously [20] to avoid bacterial translocation and interruption of bowel peristalsis. After the distal portion of the tumor was dissected, an appendicostomy was performed with a catheter inserted into the appendiceal stump. The colon was irrigated with more than three liters of saline until the proximal colon was completely cleansed, which was followed by one-stage anastomosis.

### 2.4. SEMS with Elective Surgery

Stent placement was performed by an endoscopist who had previously performed more than 400 endoscopic retrograde cholangiopancreatography (ERCP) procedures [20]. Each patient was placed with an uncovered WallFlex enteral colonic stent (Boston Scientific Corporation, Natick, MA, USA) with midbody and proximal flange diameters of 22/27 and 25/30 mm, individually, and the lengths of the stents range from 6 to 12 cm. Once the colonoscope approached the proximal tumor site, a stiffness guide wire was inserted through the narrow space of the tumor, followed by a pusher assembled out of metal stents. The stent was slowly freed at an appropriate site and fully expanded, with stent patency monitored by endoscopy. If stent placement was successful and the intestinal obstruction was relieved, elective surgery was later performed. If the surgery was unsuccessful, ES with intraoperative irrigation or manual decompression was subsequently performed.

### 2.5. Definition of Variants

Left-sided colon cancers were defined as locations including the splenic flexure, descending colon, sigmoid colon, and distal intestine up to 10 cm from the anal verge. The pathological tumor stage was diagnosed according to the American Joint Committee on Cancer (AJCC), Cancer Staging Manual, 7th edition [17]. Hypertension, diabetes mellitus, and single- and multiple-organ dysfunction were defined as comorbidities.

Perioperative complications were subdivided into five grades according to the Clavien-Dindo classification system [21, 22]. Grade I was defined as complications not requiring additional interventions or only minor interventions such as fasting; grade II was defined as complications requiring pharmacologic or other further treatments, such as blood transfusion, total parenteral nutrition, and prolonged tube feeding; grade III was defined as complications requiring surgical intervention or other interventional treatments (e.g., percutaneous drainage); grade IV was defined as life-threatening complications, including central nervous system, cardiac, and pulmonary complications, renal failure, and those requiring intensive care unit (ICU) management; grade V was defined as death.

### 2.6. Follow-Up Visits and Outcomes

All patients were followed up 1 month after surgery, every subsequent 3 months during the first postoperative year, and every 6 months thereafter until 36 months after surgery or until death. At each follow-up visit, routine blood tests, serum CEA level, and computed tomography (CT) were performed. Outcomes were overall survival (OS) and disease-free survival (DFS). Of the 72 included patients, 69 (95.83%) accept follow-up visit and 3 (4.17%) were lost as they were involuntary to follow-up visit.

### 2.7. Propensity Score Analysis

Propensity scores for all patients were estimated via a logistic regression model, which consisted of all covariates that might have affected patient short-term and survival outcomes (Figure 1). One-to-one nearest-neighbor matching was performed between the ES and SEMS groups using a 0.2 caliper width. The resulting score-matched pairs were used in subsequent analyses as indicated.

### 2.8. Statistical Analysis

Between-group differences in qualitative variables were compared using the chi-squared test or Fisher's exact test, and quantitative variables were compared using *t*-tests. As the T4 stage could be definitively recognized preoperatively via a CT scan, the sensitivity and specificity were both nearly 100%. Thus, based on the N stage and M stage [23], we transformed the multihierarchical variables, including the pT stage, pN stage, and cM stage, into binary variables. The 3-year OS and 3-year DFS were calculated by the Kaplan-Meier method. Independent risk factors for 3-year DFS and 3-year OS were analyzed using a Cox proportional hazard regression model. A stratification log-rank test was used to compare the differences between subgroups. All *p* values less than 0.05 were considered statistically significant. All statistical analyses and graphs were generated using SPSS 23.0 software.

## 3. Results

### 3.1. Baseline Characteristics of Patients

Of 162 patients who underwent surgery for OCC at our center between January 2008 and October 2014, 84 with OLCC were recruited in this study according to the strict selection criteria. Of these 84 OLCC patients, 44 underwent ES with intraoperative irrigation or manual decompression and the other 40 underwent SEMS placement as a BTS. Thirty-six patients were included in each group. After PSM, clinical parameters, including age, sex, ASA grades, pT stage, pN stage, and cM stage, as well as the location of the tumor and the rates of adjuvant chemotherapy, were precisely compared between the 2 groups (Table 1).

### 3.2. Surgery-Related Outcomes

Stent-related adverse events occurred in 11 of 36 (30.6%) patients in the SEMS group, including 6 (16.7%) with difficulty in positioning the guidewire in the lumen of the tumor, 3 (8.3%) with pathologically verified microperforation after stent placement, and 2 (5.6%) with clinical reobstruction (Table 2). The average interval was 10.07 days from insertion of the SEMS to the subsequent elective surgery.

Increased surgical time (229.0 ± 60.8 min vs 216.4 ± 70.5 min) and blood loss (233.6 ± 358.6 ml vs 161.9 ± 245.9 ml) were observed in the ES group, but the difference was not significant (*p* = 0.422 and 0.326). Stoma construction was performed in 10 (27.8%) and 6 (16.7%) cases in the SEMS and ES groups, respectively, due to insufficient bowel preparation and stent-related perforation. The postoperative complication and 30-day mortality rates, number of harvested lymph nodes, and the length of the hospital stay and gastrointestinal recovery were similar between the two groups (Table 3).

### 3.3. Risk Factors for Oncological and Survival Outcomes

**Figure 2:**
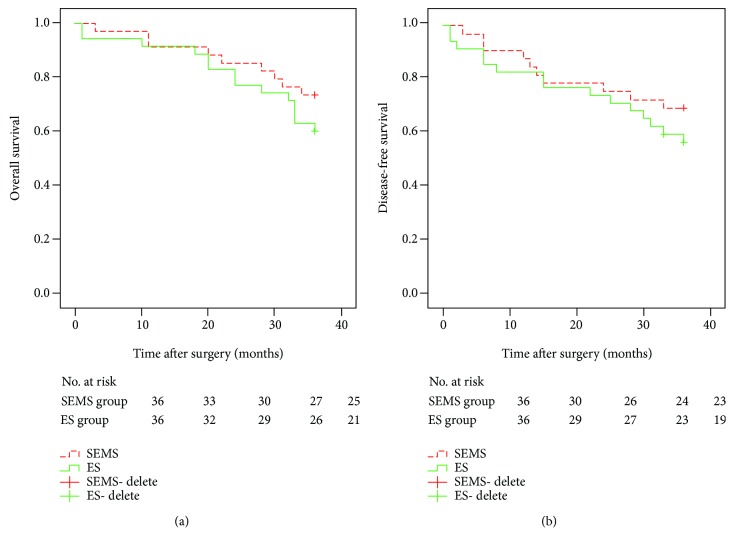
Overall (a) and disease-free survival (b) after surgery between the SEMS and ES groups. *p* > 0.05 (log-rank test).

The 3-year OS and 3-year DFS were analyzed for all patients enrolled in this study. The 3-year OS was not significantly different between the SEMS (73.5%) and ES (60.0%) groups (*p* = 0.261, Figure 2(a)). Analogously, the 3-year DFS in the SEMS (69.7%) group was similar to that of the ES (57.1%) group (*p* = 0.314, Figure 2(b)). For convenient preoperative evaluation, we transformed the pT stage into a binary covariable (pT4 and non-pT4) and the pN stage was recorded as pN0 or pN+.  Table 4 presents the univariate and multivariate analyses of 3-year DFS and 3-year OS in all OLCC patients. Only the pT stage was confirmed as an independent risk factor for 3-year DFS (*p* = 0.004). Similarly, the non-pT4 group (82.5%) was associated with higher 3-year OS rates than the pT4 group (44.8%, *p* = 0.002). Subgroup analysis of the SEMS group regarding 3-year DFS and 3-year OS also demonstrated that the pT stage was a unique risk factor for both 3-year DFS (*p* = 0.014) and 3-year OS (*p* = 0.010) (Table 5). However, the comorbidity status (*p* = 0.049) independently affected 3-year DFS in the ES group. In the ES group, the 3-year OS rate was significantly lower in the cM1 group (65.6%) than in the cM0 group (0.0%, *p* = 0.003) (Table 6).

### 3.4. Stratification Analysis of Oncological and Survival Outcomes

**Figure 3:**
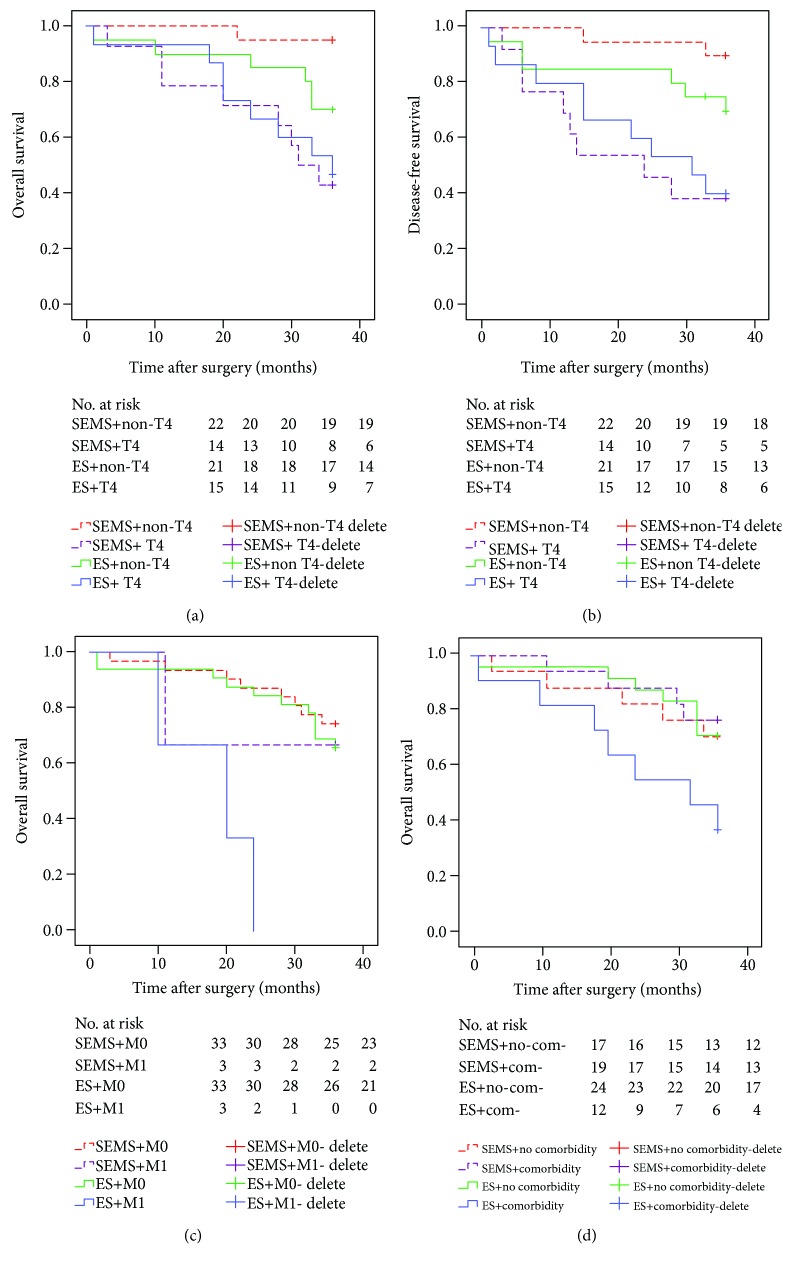
Kaplan-Meier stratification survival curves for patients in the SEMS and ES groups according to different parameters (log-rank test). (a) 3-year OS in different pT categories. *p* values for different comparisons, SEMS + non − T4 : ES + non − T4 (*p* = 0.043); (b) 3-year DFS in different pT categories. *p* values for different comparisons, SEMS + non − T4 : ES + non − T4 (*p* = 0.108); (c) 3-year OS in different cM categories. *p* values for different comparisons, SEMS + cM0 : ES + cM0 (*p* = 0.504), SEMS + cM1 : ES + cM1 (*p* = 0.197); (d) 3-year OS for different com (comorbidity) status. *p* values for different comparisons.

Due to the discrepancy in risk factors for 3-year DFS and 3-year OS in the 2 groups, stratification analysis was performed in OLCC patients. Subgroup analysis of the pT stage resulted in differences in the 3-year DFS rate and 3-year OS rate between the SEMS group and ES group. Patients with non-pT4 stages in the SEMS group showed significantly better 3-year OS (95.0%) than those in the ES group (70.0%, *p* = 0.043), but the 3-year DFS (90.0%) in the SEMS group was similar to that of the ES group (70.0%). Conversely, the estimated 3-year DFS was 38.5% and the 3-year OS was 42.7% in pT4-stage patients in the SEMS group. The 3-year DFS (40.0%) and 3-year OS (46.7%) were similar in the ES group (Figures 3(a) and 3(b)). The 3-year OS rate was 36.4% in the ES group for patients with severe comorbidities, which is significantly worse than the OS rate of the other subgroups (*p* = 0.027, *p* = 0.030, and *p* = 0.033) (Figure 3(d)). No obvious difference was observed between the SEMS and ES groups for the 3-year OS rate of patients with the same cM stage (*p* = 0.504) (Table 7, Figure 3(c)).

## 4. Discussion

This study reveals similar 3-year DFS and 3-year OS rates between the SEMS and ES groups. The independent risk factors included the status of metastasis and comorbidities in the ES group and the pT stage in the SEMS group. Through stratification survival analysis, we determined that only certain subgroups of OLCC patients would benefit from SEMS placement or ES, which might explain why controversial data have been presented in recent studies.

Concerning the negative oncological outcome in the further analysis of the SEMS group, macroscopic and microscopic perforations have been described in several studies, partly due to the stiffness of the guide wire and older age of patients [13, 18]. Two recent Dutch stent-in trials presented a high incidence of stent-related perforation (23.07%), similar to that of our center, in which 30.6% experienced stent-related adverse effects, including 6 (16.7%) with difficulty in positioning the guidewire, 3 (8.3%) with pathological microperforation, and 2 (5.6%) with reobstruction. A higher recurrence rate was shown in the perforation subgroup, and consequentially, patients without risk of perforation exhibited similar survival outcomes from either ES or SEMS placement as a BTS [15]. A high-volume center in France defined pT4 status and tumor size as independent risk factors for perforation, perineural invasion, and lymph node metastasis [13]. Consistent with previous studies, we confirmed that only non-pT4 patients in the SEMS group achieved obviously improved 3-year OS (95.0%) compared to the other subgroups but the 3-year DFS (90.0%) was similar to its counterparts. It is thought that the mechanical dilation of the stent on the colonic wall can dramatically enhance local infiltration and the dissemination of cancer into the peripheral circulation. Via circulating tumor cell detection, a Japanese study has confirmed that cancer cells would be pushed into the surrounding vessels and peripheral bloodstream by a locally stent mechanical stimulus [24, 25], which might account for the worse survival rates in pT4 patients after stent placement.

The operative risk of patients in the ES group and the concern for oncological outcomes in the SEMS group were areas of focus. The high risk of intraoperative complications and postoperative mortality were considered more threatening than the risk of stent-related perforation [26, 27]. After weighing the advantages and disadvantages, the ESGE recommended that only OLCC patients in the “high-risk” group aged > 70 years and/or with ASA scores greater than 3 should be indicated for SEMS placement [18]. A Markov Chain Monte Carlo Decision Analysis revealed that SEMS placement was more effective and less costly than ES for the treatment of OLCC and was preferred for the “low-risk” group that did not have an increased risk of stent placement failure and perforation, which would diminish its benefits. In contrast, ES was a liability to patients without increased operative risk [12]. In our center, compared to the SEMS group, the comorbidity status significantly decreased the 3-year OS rate (36.4%) in the ES group (*p* = 0.030), which might be due to the increased risk of severe perioperative complications. Similar to the positive status of metastasis in the ES group, a worse survival rate (0.0%) was observed in this subgroup; however, the survival rate was not significantly different for the corresponding subgroup of the SEMS group (*p* = 0.504).

The volume of the center greatly affects the efficacy of SEMS placement and ES for OLCC patients [28]. For some centers with highly skilled endoscopists, a high success rate of insertion and an extremely low frequency of stent failure and related complications make SEMS placement the preferred choice. For the remaining centers without distinguished endoscopists, ES is regarded as the standard procedure for OLCC treatment. In our center, the endoscopist who performed SEMS had performed more than 400 ERCP procedures, which ensured the safety and efficacy of our clinical trials.

However, this is a PSM analysis study of a cohort at one center, and thus, a prospective multicenter study should be performed in the future. In addition, the sample size was not large and additional samples are required in future research. Accounting for the above limitations, this study suggests a similar survival benefit for SEMS placement as a BTS and ES in patients with OLCC. Specifically, patients in the high-operative risk group with existing severe comorbidities could acquire more survival benefits from the SEMS strategy. As a supplement for the latest ESGE guideline, the indication for the use of SEMSs in OLCC patients may be elaborated to patients without locally advanced invasion such as the non-pT4-stage status.

## 5. Conclusion

The findings of the present study suggest that SEMS might be preferred for patients of obstructive left-sided colorectal cancer in the “high-operative risk group” with existing comorbidities or those without locally advanced invasion, such as non-pT4-stage status.

## Figures and Tables

**Table 1:** Clinical characteristics of patients.

	Before propensity matching			After propensity matching		
	SEMS	ES		SEMS	ES	
Characteristics	(*n* = 40)	(*n* = 44)	*p*	(*n* = 36)	(*n* = 36)	*p*
Age, *n* (%)			0.932			1.000
<60 y	16 (40.0)	18 (40.9)		15 (41.7)	15 (41.7)	
≥60 y	24 (60.0)	26 (59.1)		21 (58.3)	21 (58.3)	
Sex, *n* (%)			0.770			0.804
Men	27 (67.5)	31 (70.5)		23 (63.9)	24 (66.7)	
Women	13 (32.5)	13 (29.5)		13 (36.1)	12 (33.3)	
ASA grade, *n* (%)			0.434			0.772
<III	32 (80.0)	32 (72.7)		28 (77.8)	29 (80.6)	
≥III	8 (20.0)	12 (27.3)		8 (22.2)	7 (19.4)	
pT stage, *n* (%)			0.421			0.810
T1-3	22 (55.0)	28 (63.6)		22 (61.1)	21 (58.3)	
T4	18 (45.0)	16 (36.4)		14 (38.9)	15 (41.7)	
pN stage, *n* (%)			0.165			0.437
N0	9 (22.5)	16 (36.4)		9 (25.0)	12 (33.3)	
N1-2	31 (77.5)	28 (63.6)		27 (75.0)	24 (66.7)	
cM stage, *n* (%)			0.226			1.000
M0	34 (85.0)	41 (93.2)		33 (91.7)	33 (91.7)	
M1	6 (15.0)	3 (6.8)		3 (8.3)	3 (8.3)	
pTNM stage			0.303			0.718
I	0 (0.0)	2 (4.5)		0 (0.0)	2 (5.6)	
II	8 (20.0)	12 (27.3)		8 (22.2)	8 (22.2)	
III	26 (65.0)	27 (61.4)		25 (69.4)	23 (63.9)	
IV	6 (15.0)	3 (6.8)		3 (8.4)	3 (8.4)	
Lymph nodes, counts	20.03 ± 7.82	20.18 ± 7.82	0.939	20.47 ± 8.05	20.44 ± 11.56	0.991
Location of tumor, *n*			0.601			0.456
Splenic colon	5 (12.5)	10 (22.7)		5 (13.9)	10 (27.8)	
Descending colon	9 (22.5)	10 (22.7)		9 (25.0)	9 (25.0)	
Sigmoid colon	22 (55.0)	19 (43.2)		18 (50.0)	15 (41.7)	
Rectum	4 (10.0)	5 (11.4)		4 (11.1)	2 (5.6)	
Adjuvant chemotherapy, *n*	20 (50.0)	28 (63.6)	0.207	18 (50.0)	23 (63.9)	0.234
Comorbidities			0.054			0.096
With	22 (55.0)	15 (34.1)		19 (52.8)	12 (33.3)	
Without	18 (45.0)	29 (65.9)		17 (47.2)	24 (66.7)	

SEMS: self-expanding metal stents; ES: emergency surgery. All *p*values < 0.05 were considered statistically significant.

**Table 2:** Characteristics of patients with stent-related adverse events in the SEMS group.

No.	Adverse events	pT stage	Location	Stoma	Treatment
1	Failure	4	Descending colon	None	Emergency surgery
2	Failure	4	Sigmoid colon	Construction	Emergency surgery
3	Failure	3	Sigmoid colon	Construction	Emergency surgery
4	Failure	4	Descending colon	None	Emergency surgery
5	Failure	4	Descending colon	Construction	Emergency surgery
6	Failure	3	Descending colon	Construction	Emergency surgery
7	Microperforation	4	Sigmoid colon	None	Conservative
8	Reobstruction	4	Sigmoid colon	Construction	Change schedule
9	Microperforation	3	Sigmoid colon	Construction	Conservative
10	Reobstruction	3	Sigmoid colon	Construction	Change schedule
11	Microperforation	4	Sigmoid colon	None	Conservative

**Table 3:** Comparison of surgical- and pathological-related outcomes between the SEMS and ES groups.

Characteristics	SEMS (*n* = 36)	ES (*n* = 36)	*p* value
Surgical time (mins)	216.4 ± 70.5	229.0 ± 60.8	0.422
Blood loss (ml)	161.9 ± 245.9	233.6 ± 358.6	0.326
Number of LNs (*n*)	20.5 ± 8.1	20.4 ± 11.6	0.991
Time to flatus (days)	3.6 ± 1.4	3.9 ± 1.4	0.278
Time to semifluid (days)	8.7 ± 4.0	8.8 ± 4.2	0.931
Total hospital stay (days)	21.8 ± 6.9	21.8 ± 9.1	0.988
Stoma construction, *n* (%)	10 (27.8)	6 (16.7)	0.257
CD classification system, *n* (%)			1.000
Grade I	11 (30.6)	11 (30.6)	
Grade II	15 (41.7)	14 (38.9)	
Grade III	8 (22.2)	8 (22.2)	
Grade IV	2 (5.6)	3 (8.3)	
Incision infection, *n* (%)	6 (16.7)	6 (11.1)	0.496
ICU intervention, *n* (%)	2 (5.6)	1 (2.8)	1.000
30 days-mortality, *n* (%)	0 (0.0)	1 (2.8)	1.000
Histology, *n* (%)			0.659
Well differentiated	0 (0.0)	1 (2.8)	
Moderate differentiated	26 (72.2)	24 (66.7)	
Poorly differentiated	1 (2.8)	3 (8.3)	
Signet ring	9 (25.0)	8 (22.2)	
Stent related adverse events, *n* (%)	11 (30.6)		
Failure	6 (54.5)		
Perforation	3 (27.3)		
Reobstruction	2 (18.2)		
Surgical intervals (days)	10.07		

LN: lymph node; SEMS: self-expanding metal stents; ES: emergency surgery. All *p*values < 0.05 were considered statistically significant.

**Table 4:** Univariate and multivariate analyses of risk factors on prognosis in OLCCs.

OLCCs	3-year DFS				3-year OS			
	Univariate		Multivariate		Univariate		Multivariate	
Characteristic	Hazard ratio (95% CI)	*p*	Hazard ratio (95% CI)	*p*	Hazard ratio (95% CI)	*p*	Hazard ratio (95% CI)	*p*
Age (≥60 vs <60 years)	1.90 (0.64, 5.68)	0.251	—	—	1.75 (0.62, 4.92)	0.288	—	—
Sex (male vs female)	1.13 (0.42, 3.01)	0.813	—	—	0.52 (0.19, 1.44)	0.205	—	—
ASA (grade ≥ III vs grade < III)	0.75 (0.20, 2.88)	0.673	—	—	1.34 (0.42, 4.22)	0.622	—	—
Group (ES vs SEMS)	1.34 (0.51, 3.51)	0.550	—	—	1.58 (0.66, 3.81)	0.308	—	—
pT stage (pT4 vs pT1-3)	3.12 (1.15, 8.48)	0.026	3.88 (1.54, 9.77)	0.004	3.64 (1.31, 10.11)	0.013	4.04 (1.66, 9.86)	0.002
pN stage (pN+ vs pN0)	1.60 (0.49, 5.24)	0.441	—	—	1.14 (0.38, 3.44)	0.811	—	—
Metastasis (cM1 vs cM0)	—	—	—	—	2.70 (0.86, 8.47)	0.088	—	—
Comorbidity (with vs without)	1.41 (0.50, 4.00)	0.522	—	—	0.95 (0.33, 2.71)	0.918	—	—

All factors where the *p* value of univariate analysis is lower than 0.10 were involved in multivariate analysis; vs: versus; pN+ includes pN1-2; ASA grade ≥ III comprises graded IV and V; with comorbidities: defined as hypertension, diabetes mellitus, and single- and multiple-organ dysfunction.

**Table 5:** Univariate and multivariate analyses of risk factors on prognosis in the SEMS group.

SEMS group	3-year DFS				3-year OS			
	Univariate		Multivariate		Univariate		Multivariate	
Characteristic	Hazard ratio (95% CI)	*p*	Hazard ratio (95% CI)	*p*	Hazard ratio (95% CI)	*p*	Hazard ratio (95% CI)	*p*
Age (≥60 vs <60 years)	4.98 (0.52, 47.47)	0.163	—	—	1.68 (0.35, 8.00)	0.514	—	—
Sex (male vs female)	5.88 (0.24, 142.85)	0.276	—	—	1.05 (0.11, 9.98)	0.968	—	—
ASA (grade ≥ III vs grade < III)	12.66 (0.73, 218.60)	0.081	—	—	7.97 (0.84, 75.78)	0.071	—	—
pT stage (pT4 vs pT1-3)	47.51 (2.10, 1075.38)	0.015	7.54 (1.51, 37.70)	0.014	47.04 (3.34, 663.62)	0.004	15.42 (1.92, 123.79)	0.010
pN stage (pN+ vs pN0)	8.12 (0.32, 204.56)	0.203	—	—	5.45 (0.43, 69.34)	0.192	—	—
Metastasis (cM1 vs cM0)	—	—	—	—	1.35 (0.09, 20.91)	0.832	—	—
Comorbidity (with vs without)	0.54 (0.10, 3.04)	0.483	—	—	0.24 (0.05, 1.21)	0.083	—	—

All factors where the *p* value of univariate analysis is lower than 0.10 were involved in multivariate analysis; vs: versus; pN+ includes pN1-2; ASA grade ≥ III comprises grades IV and V; with comorbidities: defined as hypertension, diabetes mellitus, and single- and multiple-organ dysfunction.

**Table 6:** Univariate and multivariate analyses of risk factors on prognosis in the ES group.

ES group	3-year DFS				3-year OS			
	Univariate		Multivariate		Univariate		Multivariate	
Characteristic	Hazard ratio (95% CI)	*p*	Hazard ratio (95% CI)	*p*	Hazard ratio (95% CI)	*p*	Hazard ratio (95% CI)	*p*
Age (≥60 vs <60 years)	1.61 (0.33, 7.98)	0.559	—	—	2.18 (0.49, 9.68)	0.304	—	—
Sex (male vs female)	1.11 (0.33, 3.80)	0.867	—	—	0.61 (0.16, 2.36)	0.477	—	—
ASA (grade ≥ III vs grade < III)	0.20 (0.02, 1.76)	0.148	—	—	0.67 (0.16, 2.84)	0.588	—	—
pT stage (pT4 vs pT1-3)	2.01 (0.52, 7.71)	0.311	—	—	1.34 (0.39, 4.57)	0.644	—	—
pN stage (pN+ vs pN0)	0.75 (0.12, 4.74)	0.756	—	—	0.43 (0.10, 2.28)	0.325	—	—
Metastasis (cM1 vs cM0)	—	—	—	—	4.15 (0.84, 20.61)	0.082	8.92 (2.08, 38.18)	0.003
Comorbidity (with vs without)	4.14 (0.88, 19.55)	0.073	3.13 (1.01, 9.75)	0.049	3.24 (0.59, 17.85)	0.177	—	—

All factors where the *p* value of univariate analysis is lower than 0.10 were involved in multivariate analysis; vs: versus; pN+ includes pN1-2; ASA grade ≥ III comprises grades IV and V; with comorbidities: defined as hypertension, diabetes mellitus, and single- and multiple-organ dysfunction.

**Table 7:** Stratification analysis of oncological and survival outcomes on prognosis in OLCCs.

OLCCs with pT stage	SEMS + non − T4 (3-year DFS)			SEMS + non − T4 (3-year OS)	
	Rates (95% CI)	*p*	OLCCs with pT stage	Rates (95% CI)	*p*

SEMS + T4 (*n* = 14)	0.39 (0.25, 0.52)	0.001	SEMS + T4 (*n* = 14)	0.43 (0.30, 0.56)	0.001
ES + non-T4 (*n* = 21)	0.70 (0.59, 0.80)	0.108	ES + non-T4 (*n* = 21)	0.70 (0.60, 0.80)	0.043
ES + T4 (*n* = 15)	0.40 (0.27, 0.53)	0.001	ES + T4 (*n* = 15)	0.47 (0.34, 0.60)	0.001
SEMS + non-T4 (*n* = 22)	0.90 (0.83, 0.97)	—	SEMS + non-T4 (*n* = 22)	0.95 (0.90, 0.99)	—

OLCCs with comorbidity	ES + comorbidity (3-year OS)			ES + cM1 (3-year OS)	
	Rates (95% CI)	*p*	OLCCs with cM stage	Rates (95% CI)	*p*

ES + no comorbidity (*n* = 24)	0.71 (0.62, 0.80)	0.027	ES + cM1 (*n* = 3)	—	—
SEMS + comorbidity (*n* = 19)	0.77 (0.66, 0.87)	0.030	SEMS + cM0 (*n* = 33)	0.74 (0.66, 0.82)	0.001
SEMS + no comorbidity (*n* = 17)	0.71 (0.60, 0.82)	0.033	SEMS + cM1 (*n* = 3)	0.67 (0.40, 0.94)	0.197
ES + comorbidity (*n* = 12)	0.36 (0.22, 0.51)	—	ES + cM0 (*n* = 33)	0.66 (0.57, 0.74)	0.001

All *p* values were analyzed via log-rank test (Mantel-Cox).

## Data Availability

The data used to support the findings of this study are available from the corresponding author upon request.
